# P-1885. Continuous Infectious Diseases Coverage by Merging with a Hospitalist Practice in a Small Community Hospital System

**DOI:** 10.1093/ofid/ofae631.2046

**Published:** 2025-01-29

**Authors:** Michael Wang, Kristina Aleksoniene, Jason Tompkins, Richard Douce, John Froggatt, Mark Harrison

**Affiliations:** Corewell Health Lakeland, Saint Joseph, Michigan; Corewell Health South ; Lakeland, Burr Ridge, Illinois; Marshfield Clinic, Marshfield, Wisconsin; Michigan State University, Stevensville, Michigan; Corewell Health Lakeland, Saint Joseph, Michigan; Corewell Health Lakeland, Saint Joseph, Michigan

## Abstract

**Background:**

There is a severe shortage of infectious diseases (ID) physicians in the United States, with > 80% counties without an ID physician. Insufficient compensation is a contributing factor, with many internal medicine graduates practicing as hospitalists. Based on community needs, we merged the ID practice into the hospitalists group to create an ID-Hospitalist position.

**Figure:**
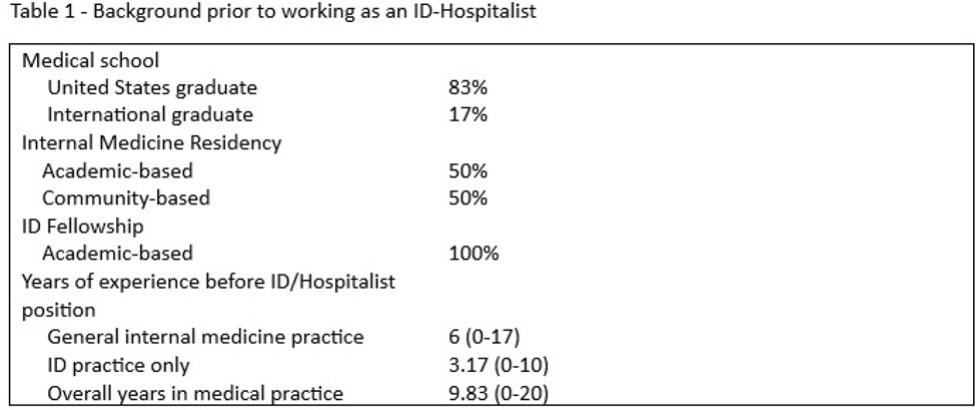

**Methods:**

Corewell Health South is based in Berrien County, which is in Southwest Michigan, population 152,261, and includes large rural populations and several federally funded clinics. The hospital includes 2 acute care facilities, including 220-bed and 76 bed hospitals. In 2002, the ID physicians began seeing patients as hospitalists at a Long-Term Acute Care Hospital. An ID physician assumed the role of Hospitalist Director in 2005 and second physician became co-director in 2008. At that time, the ID service adopted a 7 day on-7 day off schedule and included primary hospitalist patients.

**Figure:**
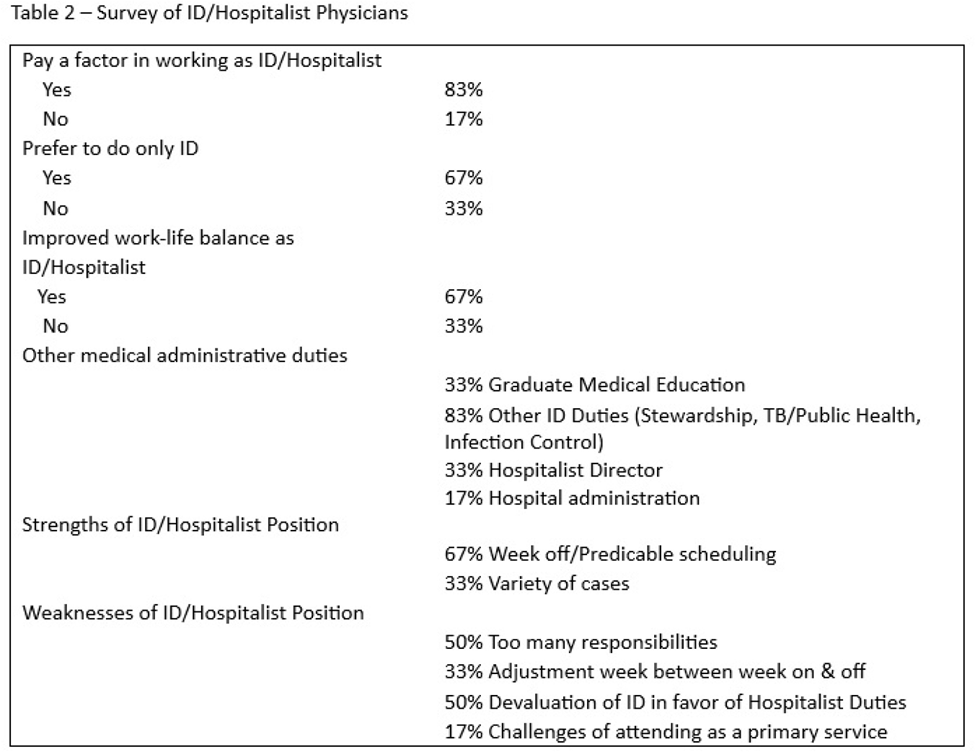

**Results:**

Since 2011, the ID-Hospitalist group has had continuous coverage with at least 3 Full time and 1 part-time ID-Hospitalists. Coverage (2.63/100,000) exceeds the national average of 1.79/100,000 by 84%. All service weeks are based on a 7 day on-7 day off schedule. In 2013, the outpatient ID clinic was resumed after a 5-year hiatus and is Ryan White Funded. A clinic day is built into the 7-day work week. Over the last 22 years, the group has included 6 ID physicians, which include 5 American graduates and 1 international graduate. In surveying the group, 83% expressed that pay was a factor in working as an ID/Hospitalist. 67% reported more free time and improved work-life balance. 67% preferred doing ID only, although the majority enjoyed hospitalist practice.

Perceived weaknesses included overextension of responsibilities and a de-emphasis on the ID service in favor of hospitalist responsibilities. Another limitation included inconsistent coverage at the smaller hospital, increasing the reliance on telephone consultations.

**Conclusion:**

Merging an ID practice into a hospitalist group has been effective in sustaining an ID service. Strengths included stable salary support and ability to perform other administrative or clinical tasks. Weakness included a multitasking of duties. This may serve as a model for resource limited settings.

**Disclosures:**

All Authors: No reported disclosures

